# Heuristics Facilitates the Evolution of Transitive Inference and Social Hierarchy in a Large Group

**DOI:** 10.1007/s10441-023-09459-5

**Published:** 2023-03-03

**Authors:** Kazuto Doi, Mayuko Nakamaru

**Affiliations:** Department of Technology and Innovation Management, Tokyo Institute of Technology, CIC-901S, 3-3-6 Shibaura, Minato-Ku, Tokyo, 108-0023 Japan

**Keywords:** Evolutionary simulation, Group size, Heuristics, Reference transitive inference, Social complexity hypothesis, Collective memory

## Abstract

Transitive inference (TI) refers to social cognition that facilitates the discernment of unknown relationships between individuals using known relationships. It is extensively reported that TI evolves in animals living in a large group because TI could assess relative rank without deducing all dyadic relationships, which averts costly fights. The relationships in a large group become so complex that social cognition may not be developed adequately to handle such complexity. If members apply TI to all possible members in the group, TI requires extremely highly developed cognitive abilities especially in a large group. Instead of developing cognitive abilities significantly, animals may apply simplified TI we call reference TI in this study as heuristic approaches. The reference TI allows members to recognize and remember social interactions only among a set of reference members rather than all potential members. Our study assumes that information processes in the reference TI comprises (1) the number of reference members based on which individuals infer transitively, (2) the number of reference members shared by the same strategists, and (3) memory capacity. We examined how information processes evolve in a large group using evolutionary simulations in the hawk–dove game. Information processes with almost any numbers of reference members could evolve in a large group as long as the numbers of shared reference member are high because information from the others’ experiences is shared. TI dominates immediate inference, which assesses relative rank on direct interactions, because TI could establish social hierarchy more rapidly applying information from others’ experiences.

## Introduction

### Background

How to increase chances of winning competitions for limited resources is critical for animals living in groups (Austad 1983; Enquist and Leimar 1983; Milinski and Parker 1991) because of significant impacts to the ability to survive and reproduce, or fitness. Previous studies on the evolution of animal conflicts examined which types of assessment of fighting ability, or resource-holding potential (RHP), of the opponent can evolve under different social conditions (e.g., Enquist and Leimar 1983; Hsu et al. 2006; Parker 1974; Reichert and Quinn 2017). Many early experimental studies assumed that body size, body mass, body color, voices and development of weaponry represent RHP. However, RHP is, sometimes, so invisible or intangible that sounds and colors are not useful at all, which is more often the case with the human society. When RHP is intangible, it is reasonable to assume that individuals can use outcomes from social interactions, such as wins or losses in animal contests, as indicators of RHP. Social interactions in animal contests play critical roles in forming the social hierarchy (Arnott and Elwood 2009; Hsu et al. 2006; Reichert and Quinn 2017).

There are many previous theoretical studies (e.g., Chase 1982; Dugatkin 1997, 2001; Dugatkin and Earley 2003; Nakamaru and Sasaki 2003) about types of assessment of RHP and the formation of the social hierarchy. Lindquist and Chase (2009) found that the winner-loser effect, defined as an increased probability of winning on past victories and an increased probability of losing on past defeats, does not show satisfactory agreement with the hen data they analyzed and suggested that individuals in a group are intensively aware of interactions among other members in their group.

Huang and Wu (2022) looked into the relationship between hierarchical structures and memory capacities and finds commonly observed hierarchical structures in nature as the total fitness-maximizing social structures given different levels of cognitive abilities.

**Fig. 1:**
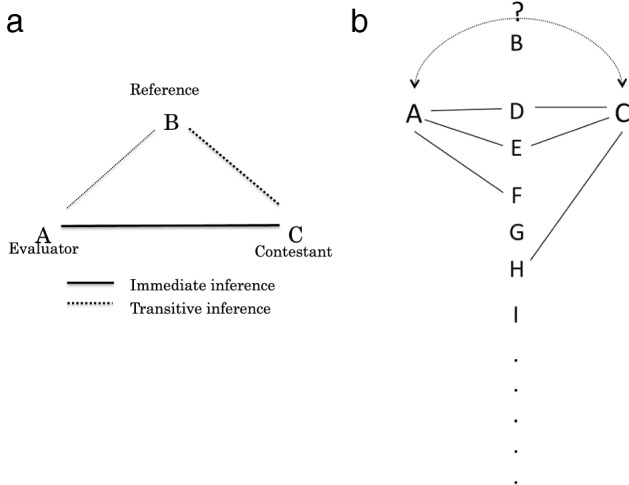
**a** Transitive inference vs. immediate inference. Player A is an evaluator and will have a contest with player C (contestant). If A has direct contests with C in the past, A can evaluate C’s strength based on the results of direct contests with C. This is called immediate inference. If A has no direct contests with C in the past but has direct contests with B (reference) who has direct contests with C, A can evaluate C’s strength transitively based on the results of contests between A and B and the ones between B and C. This is called transitive inference. **b** Transitive inference process： the number of reference members and the number of shared reference members in TI_*X–Y*_ in the case of two TI_3–2_ players in a group. Players A and C follow the TI_3–2_ strategy. Given the number of reference members = 3, solid lines show that reference members for A are D, E and F and reference members for C, are D, E and H. Players applying the TI_3–2_ strategy are assumed to share two players with the other TI_3–2_ players since the number of shared reference members is 2. Shared reference members for A and C are D and E in the present example. Reference members are randomly chosen. Dotted line shows that A and C attempt to make an assessment of the relative rank each other using transitive inference-process when there are no direct contests between A and C. Player B adopts the immediate inference strategy

Chase and Lindquist (2016) developed a theoretical approach that uses sequences of interactions with others within a group to explain the organization of the social hierarchy. They emphasized the importance of social cognition by taking eavesdropping, individual recognition and transitive inference as an example of social cognition (Hsu et al. 2006). Transitive inference is particularly important because it uses known relationships to deduce unknown ones. For example, A knows that A is stronger than B and B is stronger than C, but does not know if A is stronger than C. If A can have the ability of transitive inference, A can infer A > C, using A > B and B > C (Fig. 1a). Social cognition allows an individual to identify others, recognize and remember its relationship with others (Bshary and Brown 2014; Seyfarth and Cheney 2003).

### Transitive Inference and the Social Complexity Hypothesis

The social complexity hypothesis suggest that complex societies may promote the evolution of social cognition because members need to be able to handle the complexity from social interactions (Balda and Kamil 1989; Bond et al. 2003, 2010; de Waal and Tyack 2003; Jolly 1966). Reichert and Quinn (2017) and Hobson (2020) pointed out the importance of cognitive mechanisms that underlie contest behaviors, which little is known about.

Increasingly more studies support the social complexity hypothesis (Balda and Kamil 1989; Bond et al. 2003, 2010; Fernald 2014, 2017; Jolly 1966; MacLean et al. 2008; de Waal and Tyack 2003). As the group size increases, the number of possible interactions between pairs of individuals dramatically increases. Therefore, in a large group, it becomes increasingly difficult to understand the social hierarchy through the understanding of dyadic relationships between pairs of individuals in a group. For example, the number of members in a group in the study on the social hierarchies in *Astatotilapia burtoni* was 20 (Fernald 2014). In this case, every member has to remember the results of interaction with other all 19 members, which takes a long time. This process takes a longer time as the group size increases. Transitive inference helps a member to assess RHP of the opponent the member has never interacted with before. Therefore, as the group size increases, transitive inference is more important. Transitive inference can assess opponents’ RHP even without remembering all of the dyadic relationship with the opponents by using information from others. Therefore, transitive inference becomes increasingly important in the context of the social complexity hypothesis (Bond et al. 2003, 2010). Transitive inference is considered as a way of facilitating the understanding of social hierarchy without increasing the direct dyadic relationship under limited memory capacity (Mikolasch et al. 2013; Paz-Y-Miño et al. 2004).

Nakamaru and Sasaki (2003) and Doi and Nakamaru (2018) studied how transitive inference evolves, related to the social hierarchy formation theoretically by using the evolutionary game theory. These studies assume that individuals play the asymmetric hawk-dove game (Parker 1974; Maynard Smith 1974; Maynard Smith and Parker 1976). This framework has often been employed in the analysis of the evolution of fighting behaviors in animals.

Nakamaru and Sasaki (2003) demonstrated that the ability to accurately assess RHP is favored when the cost of losing is slightly larger than the benefit because the hawk vs. hawk combination occurs more often with lower costs. This ability is demonstrated in a strategy referred to as immediate inference (II) strategy in Doi and Nakamaru (2018) and Nakamaru and Sasaki (2003) where a player who estimates the strength of an opponent based on the history of direct fights or dyadic interactions (Fig. 1a). In contrast, the ability to form the social hierarchy promptly would be favored more when the cost is much higher than the benefit. This ability is associated with transitive inference (TI) strategy (Doi and Nakamaru 2018; Nakamaru and Sasaki 2003).

Both types of inferences have been reported extensively in the animal kingdom (Allen 2013; Grosenick et al. 2007; Paz-Y-Miño et al. 2004; Vasconcelos 2008; White and Gowan 2013). Both immediate and transitive inferences require social cognition, which refers to information learned about the characteristics of other individuals in the social interactions or based on observations (Sheehan and Bergman 2016). However, different types of inferences require considerably different types of social cognition. For example, immediate inference requires individuals to recognize only individuals that they have interacted with while transitive inference requires individuals to recognize an individual that they have never interacted with (Bshary and Brown 2014; Lilly et al. 2019; Seyfarth and Cheney 2015).

Social cognition has been investigated extensively in a wide range of animals, including both vertebrates and invertebrates (Emery et al. 2007; Gheusi et al. 1994). In particular, a recent report that transitive inference is observed even in insects such as wasps adds evidence that the miniature nervous system of insects does not limit sophisticated social behaviors (Tibbetts et al. 2019). This looks like a puzzle suggesting that the relationship between social information use in transitive inference and cognitive abilities may not be straightforward, given that social information use is common in taxa with advanced cognitive capacity like primates (Tibbetts et al. 2022). This study aims to show one of the possible answers to this puzzle.

Doi and Nakamaru (2018), which tried to answer the puzzle, theoretically demonstrated that transitive inference evolves with relatively low memory capacity when the cost of losing is relatively high because transitive inference can form the social hierarchy promptly. Furthermore, lower memory capacity is even more effective because lower memory capacity may restore the more consistent social hierarchy with true ranking based on actual RHP by disregarding existing social hierarchy that is not necessarily consistent with RHP.

However, Doi and Nakamaru (2018) still assumed highly developed social cognition that allowed individuals to recognize any other individuals that the individual had not interacted with and remembered all the outcomes of contests among any individuals. If all members apply transitive inference to all possible members in the group, transitive inference is supposed to require extremely highly developed cognitive abilities especially in a large group because every member needs to remember results of interactions by *N* × (*N* − 1)/2 pairs. Therefore, we relax this assumption in our present study. Our hypothesis in this study is that, instead of developing cognitive abilities substantially, animals may apply a kind of simplified transitive inference we call reference transitive inference as heuristic approaches. The reference transitive inference allows members to recognize and remember social interactions only among a set of reference member rather than all potential members. Animals may apply heuristic approaches such as reference transitive inference to handle such complex scenarios, instead of developing social cognition.

### Heuristics, Collective Memory and Social Complexity

Individuals using reference transitive inference in a group infer the strength of unknown members transitively based on a set of reference members who can be a group of arbitrary members by applying information from experiences by other reference members. We also assume that the ability to share reference members with individuals following the same strategy (Fig. 1b). Sharing reference members in a group requires not only cognitive capability but also social interactions such as some form of information exchanges among reference members, similarly to collective memories that refer to the pool of memories, knowledge and information shared in a social group. Coman et al. (2016) discusses the formation of collective memories in communication networks in laboratory-created communication. Weldon (2000) explored the formation of collective memories by using social network methodology. However, we did not consider the process to form shared memories and to choose reference members, for simplicity, in this study.

We redefine transitive inference as TI_*x–y*_, where individuals can recognize and focus on an *x* number of reference members (*x* ≤ *N* − 1). Individuals following the same strategy share *y* number of members out of *x* number of reference members (*y* ≤ *x* ≤ *N* − 1). Figure 1b shows that how the number of reference members and the number of shared reference members interact in the transitive inference-process. Player A and C both employ a TI_3–2_ strategy. Considering the number of reference members = 3, we assume that the reference members for A are D, E and F, and the reference members for C, are D, E and H. Players applying the TI_3–2_ strategy are assumed to share the information of two players D and E with other TI_3–2_ players. D and E are shared reference members for all TI_3–2_ players in the group. Shared reference members would help us to understand how sharing the same information in TI promotes the formation of the social hierarchy.

The heuristic approaches in reference transitive inference could substantially reduce the number of pairs required for understanding the entire social hierarchy, compared to immediate inference, when the group size is large. For example, immediate inference needs information about *N* − 1 pairs and TI without heuristics requires information about *N* × (*N* − 1)/2 pairs, while TI_1–1_ requires only *N* − 1 relationships at minimum. Even limited number of reference members and shared reference members could facilitate the prompt establishment of the social hierarchy.

In this study, we consider the size of a group one of components influencing social complexity for simplicity. Social complexity is a common, but a little controversial, concept due to a lack of objectivity and a failure to link sociality to the application of cognition (Bergman and Beehner 2015). A review study about goldfish and parrots by Croney and Newberry (2007) and a comparative study of six primate species by MacLean et al. (2013) suggest that the group size signficantly influences the development of social cognition. However, the use of the group size as an index of social complexity is sometimes criticized because it does not take into account the diverse interactions among different animals within groups (Bergman and Beehner 2015).

## Model

### Hawk-Dove Game

We consider a group of *N* players. We pick two players, A and B, at random, from the group. A and B play the asymmetric hawk-dove game. Each player is supposed to select hawk (escalation) or dove (retreat). If both opt for dove, they share the reward *V* equally without fighting. Each receives *V*/2. If one player opts for hawk and the other player opts for dove, the hawk player wins and receives reward *V*. The dove loses and gains no reward. If both opt for hawk, they fight actually. The player who wins receives the reward *V* while the player who loses has to pay the cost, − *C* (*V*, *C* > 0). The chance that A wins against B is determined by the difference of RHP of A and B based on the function $\theta \left( {x_{A} ,{ } x_{B} } \right)$ in the Eq. (1) below.

1 $$\theta \left( {x_{A} ,{ } x_{B} } \right){ } = { }\frac{1}{{1{ } + e^{{ - \left( {x_{A} { }{-}{ }x_{B} } \right)/a}} { }}}$$

where $x_{A}$ and $x_{B}$ correspond to RHP for players A and B respectively. Equation (1) suggests that when the A’s RHP is higher than B’s, A is more likely to win. When the value of *a* is lower, the probability that a player with a higher RHP would win is higher. The classical hawk–dove game assumes that $\theta \left( {x_{A} ,{ }x_{B} } \right)$ is 1/2 regardless of RHP.

It is an evolutionarily stable strategy (ESS) that players opt for hawk with a chance of *V*/*C* when *V* < *C*, or that players always opt for hawk when *V* ≥ *C*.

### Strategies and Assumptions

#### Three Types of Inference Processes

The strategies on which players select hawk or dove are genetically determined traits. We assume three types of strategies: mixer strategy (M), immediate inference strategy (II), and transitive inference strategy (TI_*x–y*_) (Table 1).

**Table 1:** Summary of strategies

	Inference processes			Information processes		
Strategies	TI-process	II-process	Mixer-process	*x*	*y*	*MC*
M	–	–	√	–	–	–
II	–	√(1)	√(2)	0	0	14
TI_2–0_	√(2)	√(1)	√(3)	2	0	14
TI_2–2_	√(2)	√(1)	√(3)	2	2	14
TI_4–0_	√(2)	√(1)	√(3)	4	0	14
TI_4–2_	√(2)	√(1)	√(3)	4	2	14
TI_4–4_	√(2)	√(1)	√(3)	4	4	14
TI_6–0_	√(2)	√(1)	√(3)	6	0	14
TI_6–2_	√(2)	√(1)	√(3)	6	2	14
TI_6–4_	√(2)	√(1)	√(3)	6	4	14
TI_6–6_	√(2)	√(1)	√(3)	6	6	14
TI_8–0_	√(2)	√(1)	√(3)	8	0	14
TI_8–2_	√(2)	√(1)	√(3)	8	2	14
TI_8–4_	√(2)	√(1)	√(3)	8	4	14
TI_8–6_	√(2)	√(1)	√(3)	8	6	14
TI_8–8_	√(2)	√(1)	√(3)	8	8	14

The mark √ shows which inference process each strategy adopts. The number in () next √ to indicates the order of priority in inference processes. For example, when (1) is available (1) is employed and when (1) is not available (2) is employed. 1 is the highest priority order and 3 is the lowest. *MC* in information processes stands for memory capacity defined as the number of contests players can remember

We consider social cognition as a set of processes to (a) make an inference and (b) to gather and store the information for inference. The first part is referred to as inference processes while the second part is referred to as information processes (Table 1) in this study. As listed in Table 1, the strategies comprise of inference and information processes. Information processes comprise three parts: (1) The number of reference members who the focal individual can recognize and focus on, (2) The number of reference members shared by individuals (Fig. 1b), and (3) Memory capacity.

Each strategy consists of some of three types of inference processes, including mixer-process, immediate inference (II)-process, and transitive inference (TI)-process.

First, we will explain three types of inference processes. In mixer-process, a player makes a selection between hawk and dove following a mixed ESS where hawk is selected with a probability of *V*/*C* and dove with 1 − *V*/*C* when *C* (cost) ≥ *V* (reward). The player does not infer the strength of others. In addition, a player adopts the mixer-process when there is no assessment due to the lack of both ties and related contests.

We define *R*_*X*_ (B|A) as an assessment by player X of the relative rank of player B to A based on the past interactions between A and B. *R*_*X*_ (B|A) takes one of three values, 1, − 1 or 0. *R*_*X*_ (B|A) = 1 indicates that X assesses B stronger than A, if B has more wins than losses in the past contests between A and B. *R*_*X*_ (B|A) =  − 1 suggests that X assesses A stronger than B, if A has more wins than losses. *R*_*X*_ (B|A) = 0 means that X perceives A and B indifferent, if A ties with B or if there are no contests between A and B. We equally count as wins (losses) both wins (losses) in hawk vs. hawk and wins (losses) in hawk vs. dove. We consider only the signs, positive or negative, of differences of the numbers of wins and losses, not the magnitude of the differences.

In immediate inference-process, player A selects hawk when *R*_*A*_ (B|A) =  − 1 and dove when *R*_*A*_ (B|A) = 1. Similarly, player B opts for hawk when *R*_*B*_ (A|B) =  − 1 and dove when *R*_*B*_ (A|B) = 1.

With regard to the transitive inference-process, we assume that TI_*x*–*y*_ players have the ability to observe and recall all contestants and results of contests only among *x* reference members in their information set where *y* reference members out of *x* are shared among the TI_*x*–*y*_ players. The *x*–*y* components in TI_*x–y*_ represent the information processes characterized as a combination of two parts, (1) and (2) in information processes and correspond to heuristic mechanisms in transitive inference.

A set of shared reference members, referred to as *y*, is randomly determined from the group. Once *y* players are set, (*x–y*) players are selected randomly from the group. We decide to select reference members randomly from the group, not in other ways. In fact, how to select reference members could depend on the relationships and availabilities among individuals under different social settings. Such realistic ways of selecting reference members would require more perplexing assumptions including various social contexts. Therefore, the random selection of reference members allows our study to focus on the complexity by the large group size.

Let us consider player A and B who need to assess the strengths each other. They have no direct contest, but both have direct contests with player C in the past. If *R*_*A*_ (B|C) = 1, or B > C, and *R*_*A*_ (C|A) = 1, or C > A, then *R*_*A*_ (B|A) (= *R*_*A*_ (B|C) + *R*_*A*_ (C|A)) = 2 > 0, or B > A. If *R*_*A*_ (B|A) (= *R*_*A*_ (B|C) + *R*_*A*_ (C|A)) > 0, then we set *R*_*A*_ (B|A) = 1. Here, transition inference suggests that if A < C and C < B, then A < B.

Similarly, if *R*_*A*_ (B|C) =  − 1, or C > B, and *R*_*A*_ (C|A) =  − 1, or A > C, then *R*_*A*_ (B|A) (= *R*_*A*_ (B|C) + *R*_*A*_ (C|A)) =  − 2 < 0, or A > B. If *R*_*A*_ (B|A) (= *R*_*A*_ (B|C) + *R*_*A*_ (C|A)) < 0, then we set *R*_*A*_ (B|A) =  − 1. Then, transitive inference intimates that if A > C and C > B, then A > B.

If players cannot infer the strength of the opponent with transitive inference, the players follow a mixed ESS. For example, if *R*_*A*_ (B|C) = 1, or B > C and *R*_*A*_ (C|A) =  − 1, or A > C then A considers that B is as potent as A (*R*_*A*_ (B|A) = *R*_*A*_ (B|C) + *R*_*A*_ (C|A) = 0). If *R*_*A*_ (B|A) (= *R*_*A*_ (B|C) + *R*_*A*_ (C|A)) = 0, then we set *R*_*A*_ (B|A) = 0. In this case transitive inference suggests no difference between A and B.

We introduce a function *F*(*R*) defined as follows to simplify the process: *F*(*R*) = 1 (if *R* > 0), *F*(*R*) = 0 (if *R* = 0), and *F*(*R*) =  − 1 (if *R* < 0). With the function, *R*_*A*_ (B|A) can be expressed as:

2 $$R_{A} \left( {{\text{B}}|{\text{A}}} \right) = F\left( {R_{A} \left( {{\text{B}}|{\text{C}}} \right) + R_{A} \left( {{\text{C}}|{\text{A}}} \right)} \right)$$

Generally, the number of opponents in common between A and B can be 2 or more. We refer to the individual common opponents as CO_*i*_. We calculate $R_{X} ({\text{B}}|{\text{A}})$ based on each CO_*i*_. Then, transitive inference-process is defined as follows: CO_*i*_ are included in a set of players in the reference members and the maximum number of CO_*i*_ is *x*. The number of CO_*i*_ is *n*. Therefore, $R_{X} ({\text{B}}|{\text{A}})$ can be expressed as:

3 $$R_{X} \left( {{\text{B}}|{\text{A}}} \right) = F\left. {\left( {\frac{1}{n}\mathop \sum \limits_{i}^{{\text{n}}} F(R_{X} \left( {{\text{B}}|{\text{CO}}_{i} } \right) + R_{X} \left( {{\text{CO}}_{i} |{\text{A}}} \right)} \right.)} \right)$$

Using Fig. 1b, let us explain how player A and C, TI_3–2_ players, assess RHP each other. If A and C have direct contests with A’s reference members, D, E and F, player A could assess the relative rank of A to C when there are no direct contests between A and C based on Eq. (3) as follows:

$$\begin{gathered} R_{A} \left( {{\text{C}}|{\text{A}}} \right) = F\left( {1} \right./{3}\left( {\left( F \right.} \right.\left( {R_{A} \left( {{\text{C}}|{\text{D}}} \right) + R_{A} \left( {{\text{D}}|{\text{A}}} \right)} \right) + F\left( {R_{A} \left( {{\text{C}}|{\text{E}}} \right) + R_{A} \left( {{\text{E}}|{\text{A}}} \right)} \right) \\ + F\left( {R_{A} \left( {{\text{C}}|{\text{F}}} \right) \, + R_{A} \left. {\left. {\left( {{\text{F}}|{\text{A}}} \right)} \right)} \right)} \right) \\ \end{gathered}$$

If A does not have direct contests with F, *R*_*A*_ (F|A) is not available. The transitive inference-process is based on the following equation, instead of the equation above:

$$R_{{\text{A}}} \left( {{\text{C}}|{\text{A}}} \right) = F\left( {{1}/{2}\left( {F\left( {R_{{\text{A}}} \left( {{\text{C}}|{\text{D}}} \right) + R_{{\text{A}}} \left( {{\text{D}}|{\text{A}}} \right)} \right) + F\left( {R_{{\text{A}}} \left( {{\text{C}}|{\text{E}}} \right) + R_{{\text{A}}} \left( {{\text{E}}|{\text{A}}} \right)} \right)} \right)} \right)$$

Similarly, if C and A have direct contests with C’s reference members, D, E and H, player C could assess the relative rank of C to A when there are no direct contests between the two based on Eq. (3) as follows:

$$\begin{gathered} R_{C} \left( {{\text{A}}|{\text{C}}} \right) = F\left( {1} \right./{3}\left( {\left( F \right.} \right.\left( {R_{C} \left( {{\text{A}}|{\text{D}}} \right) + R_{C} \left( {{\text{D}}|{\text{C}}} \right)} \right) + F\left( {R_{C} \left( {{\text{A}}|{\text{E}}} \right) + R_{C} \left( {{\text{E}}|{\text{C}}} \right)} \right) \\ + F\left. {\left. {\left( {R_{C} \left( {{\text{A}}|{\text{H}}} \right) + R_{C} \left( {{\text{H}}|{\text{C}}} \right)} \right)} \right)} \right) \\ \end{gathered}$$

Thus, the assessment by A of relative rank of A to C through shared reference members, D and E is common with the assessment by C of relative rank of C to A. Therefore, the social hierarchies built by TI_*x–y*_ with more shared reference members will be more similar as the number of shared reference members increases.

Our assumption allows player D to be part of *y* players if D is also a TI_3–2_ strategist, because *x* and *y* are assumed to be selected from a group including the focal players. In this case, we define *R*_*D*_ (D|D) = 0. In general, *R*_*X*_ (X|X) is defined as zero when X represents a player employing the TI_*x–y*_ strategy.

In transitive inference-process, player A opts for hawk if *R*_*A*_ (B|A) < 0 and dove if *R*_*A*_ (B|A) > 0.

#### The Definition of the Strategies

The mixer strategy always employs mixer-process and does not require information about the contests. There are no reference members, no reference members shared by individuals, and no memory capacity in the information processes for the mixer strategy (Table 1).

Immediate inference strategy uses immediate inference-process basically and then mixer-process when the immediate inference-process does not produce information useful for an assessment based on information about contests the focal players directly involved. In the information process, there are no reference members and no reference members shared by individuals, and a limited memory capacity is needed for II-process (Table 1). TI_*x–y*_ strategy first relies on the immediate inference-process, shifts to the transitive inference-process when the immediate inference-process produces no useful information for an assessment and finally shifts to the mixer-process when the transitive inference-process results in no useful information (Table 1). Information processes for TI_*x*–*y*_ strategy are based on the contests by the *x* reference members where *y* reference members are shared. A memory capacity is needed for II-process and TI-process.

We focus on the situations where the group size, *N*, ranges from 10 to 50 members, large relative to the size of reference members and the cost of losing is high. This is because we consider the relative group size of cognitive abilities represented by the size of reference members, not absolute group size, is critically important in light of our research question. We defined the ranges of the number of reference members and the number of shared reference members both from 0 to 8 by 2 to facilitate the analysis of a broad range of parameters without a significant increase in computational complexity caused by an increase in the group size.

The present study employs 16 strategies in total; mixer, immediate inference, and 14 types of transitive inference strategies expressed as TI_*x*–*y*_, including TI_2–0_, TI_2–2_, TI_4–0_, TI_4–2_, TI_4–4_, TI_6–0_, TI_6–2_, TI_6–4_, TI_6–6_, TI_8–0_, TI_8–2_, TI_8–4_, TI_8–6_ and TI_8–8_. The strategies are designed to study how transitive inference evolves under the limited social cognition as defined above.

In our context, standard transitive inference, which appears in Doi and Nakamaru (2018) and Nakamaru and Sasaki (2003), is considered as TI_*N-N*_ when the group size is *N*. Standard transitive inference represents a unique case where the number of shared reference members, the number of reference members and the group size are all equal to *N*. In standard transitive inference, all players can recognize and recall all players and information about them in a group. Our study focuses on more general circumstances with the number of shared reference members ≤ the number of reference members < the group size, where players can recognize and recall only a limited number of other players in a group. TI_*x–y*_ represents more limited information processes than TI_*N-N*_ because *x* and *y* are not greater than the group size, *N*.

When the group size is smaller and closer to the number of reference members, (*x*–*y*) players are more likely to be overlapped among players with the same strategy TI_*x*–*y*_. Before making a detailed explanation, our brief conclusion is that impacts should be very marginal when the group size is greater than 10 considering that the number of reference members is equivalent to eight.

Overlapping members in a set of reference members among the same strategists in the group emerges when the number of reference members is close or equal to the group size. When the number of reference members is equal to the group size, all members in the set of reference members are identical. Therefore, all members share all reference members (*x* = *y* as a result). If a set of reference members is determined randomly from the group, assuming that the number of shared reference members is zero, we can count how many members in a set of reference members may overlap. As the number of reference members decreases to a level lower than the group size, the expected number of overlapped reference members among the same strategists, declines. For example, when the group size and the number of reference members are eight, any TI_8–*y*_ (*y* < 8) is identical to TI_8–8_. When the group size is eight and the number of reference members is seven, the number of overlapped reference members declines substantially*.*

To clarify the impacts of the overlapping, we simulated how many reference members would overlap when the group size is ten assuming that a set of reference members is each determined randomly and the number of shared reference members is zero, or TI_*w*-0_. We observe that the number of overlapped members among all members is 10 when *w* = 10; four when *w* = 9; one when *w* = 8, and zero when *w* = 7. These results suggest that such overlapping could influence TI_8–*y*_ (*y* < 8) marginally but would not affect any TI_*x–y*_ (*x* ≤ the number of reference members = 7) when the group size is 10. Therefore, we do not consider that the overlapping could influence any TI_*x–y*_ when the group size is larger than 10. Overlapping would not matter overall because we focused on a large group.

### Evolutionary Dynamics with Mutation

We consider a generation of *T* units of time. We assign a new RHP to each player at the beginning of each generation in a random manner and remains unchanged until it is reset. RHP is regarded as a nonhereditary trait expressed as a real number randomly chosen from a uniform distribution between 0 and 10, exclusive of 10. In one unit of time, two players who are randomly picked from the group play the hawk–dove game. The players opt for hawk or dove based on their strategies. After repeating the procedure *T* times in a single generation, the payoff for players is aggregated strategy by strategy. Subsequently, the number of players with the specific strategy at the start of the next generation is proportional to the aggregate payoff of players for the strategy in the prior generation. The aggregate payoff is calculated to be positive by adding an absolute value of expected minimum payoffs to all players in order to avoid negative payoffs.

We assume that mutation occurs in the following two loci with a probability of *μ* independently: one is the number of reference members, referred to as *x-*locus and the other is the number of shared reference members, referred to as *y-*locus, which does not represent a set of specific members. A set of specific members is a nonhereditary trait.

Even though mixer and immediate inference strategies do not depend on the number of reference members or the number of shared reference members, we technically assign *x* = 0 to the mixer strategy, *x* = 1 to the immediate inference strategy, and *y* = 0 to both mixer and immediate inference strategies. Then combinations of *x* and *y* are unique to each strategy so that mutation in the *x* and/or *y* loci means mutation in strategies.

We assume that mutation is allowed to occur randomly in the *x*-locus and then in the *y-*locus regardless of the current positions in the arrays. The new values in the *x*-locus and in the *y-*locus following mutation are allowed to adopt any values in the *x-*locus and the *y-*locus under *y* ≤ *x* conditions. So, *x* ∈ {0, 2, 4, 6, 8}. For each *x*, *y* ∈ {0} in *x* = 0, *y* ∈ {0, 2} in *x* = 2, *y* ∈ {0, 2, 4} in *x* = 4, *y* ∈ {0, 2, 4, 6} in *x* = 6, and *y* ∈ {0, 2, 4, 6, 8} in *x* = 8. For example, when the prevailing positions in *x-*locus and *y-*locus are 2 and 0, respectively, the new *x-*locus value following mutation could be 0, 4, 6 or 8, excluding 2, the current value, with the same probability, *μ*/4. If the new value in the *x-*locus is 8, the new *y-*locus values could be 2, 4, 6 or 8 excluding 0, the prevailing value, with the same probability, *μ*/4.

Finally, the next generation begins. This process continues over *G* generations. The group size is constant throughout a generation. Here we apply *μ* = 0.001.

### How to Measure the Social Hierarchy Formation

We introduce an analytical index modified based on consistency index (*CI*) developed in Doi and Nakamaru (2018) as an indicator of how consistency between *R*_*i*_(*j*|*i*) and *R*_*j*_(*i*|*j*) in any two players, *i* and *j*, evolves as players play games more, assuming that all players follow the same strategy in a group.

*CI*_1_ measures how the number of reference members and the number of shared reference members influences the process of establishment of social hierarchy.

Details about *CI* are discussed in Doi and Nakamaru (2018). In short, *CI* = 0 indicates that complete consensus is built where all combinations of tactics are hawk vs. dove or dove vs. hawk. Higher *CI* suggests more disagreements. The highest *CI* is 0.5, indicating complete disagreements.

In the present study we define *CI*_1_ as 1 − *CI*/0.5, where *CI*_1_ = 1 indicates perfect consensus while *CI*_1_ = 0 means no consensus. As players play games more and more, *CI*_1_ (0 ≤ *CI*_1_ ≤ 1) is expected to increase as a social hierarchy is established.

### Key Parameters

There are four key parameters characterizing social conditions including (1) group size (*N*), (2) *C*/*V* ratio, which is a cost divided by a reward, (3) *N*_*p*_ (= 2* T*/(*N* × (*N* − 1))), referring to the expected number of contests per pair of players, and 4) Memory capacity (*MC*).

Here we use *N*_*p*_ = 2 because *N*_*p*_ = 2 gives two chances of participating in a contest to any pairs on average and Doi and Nakamaru (2018) suggest that TI works well under *N*_*p*_ = 2. *N*_*p*_ = 2 means that the encounter rates remain constant regardless of the group size because we increase *T* units of time as the group size increases. We use the constant *N*_*p*_ = 2 for all analyses in the present study for simplicity.

In the present study, we consider the group size (*N*) as one of components of social complexity as discussed in Sect. 1.2.

How reliable information from contests is in assessing RHP depends on the *C/V* ratio. For example, the probability (= (*V*/*C*)^2^) that both players opt for hawk is low when *C*/*V* is high, so that results do not reflect actual RHP because the rank is often set without actual fights. The *C*/*V* ratio is a key parameter influencing what strategies can persist. We maintain the reward constant (*V* = 4) and vary the cost. We focus on the results when the cost is high (*C* = 30) because it is known that transitive inference persists in high-cost environments (Nakamaru and Sasaki 2003).

Memory capacity (*MC*) is defined as the number of contests players can remember. For example, immediate inference players maintain *MC* of records in memory about contestants and the results of their own direct contests. We assume that players forget older records beyond memory capacity and maintain only the latest *MC* of records. In the present study, we keep memory capacity constant (*MC* = 14) and then change group size in most of our analyses because we consider it reasonable to assume that memory capacity is limited (Miller 1956). The minimum memory capacity required for an individual to understand a relationship with others is *N* − 1. We consider *N* − 1 too low as a memory capacity; therefore, we set memory capacity as 2 × (*N* − 1) given *N*_*p*_ = 2. *MC* = 14 assumes that the lowest size of a group is eight. When the group size is eight, TI_8–8_ with *MC* = 14 represents adequate social cognition. This assumption means that individuals can remember 14 records of contests out of the expected numbers of encounters, 98 (= 2 × (50 − 1)), when *N* is 50. All observations in memory are treated equally. In addition, we carried out sensitivity analysis of memory capacity by changing memory capacities under a constant group size.

However, inference process in the strategies gives priority to information about direct contests by first applying immediate inference-process, which is more direct experiences and then transitive inference-process in case of no direct contests.

## Results

**Fig. 2:**
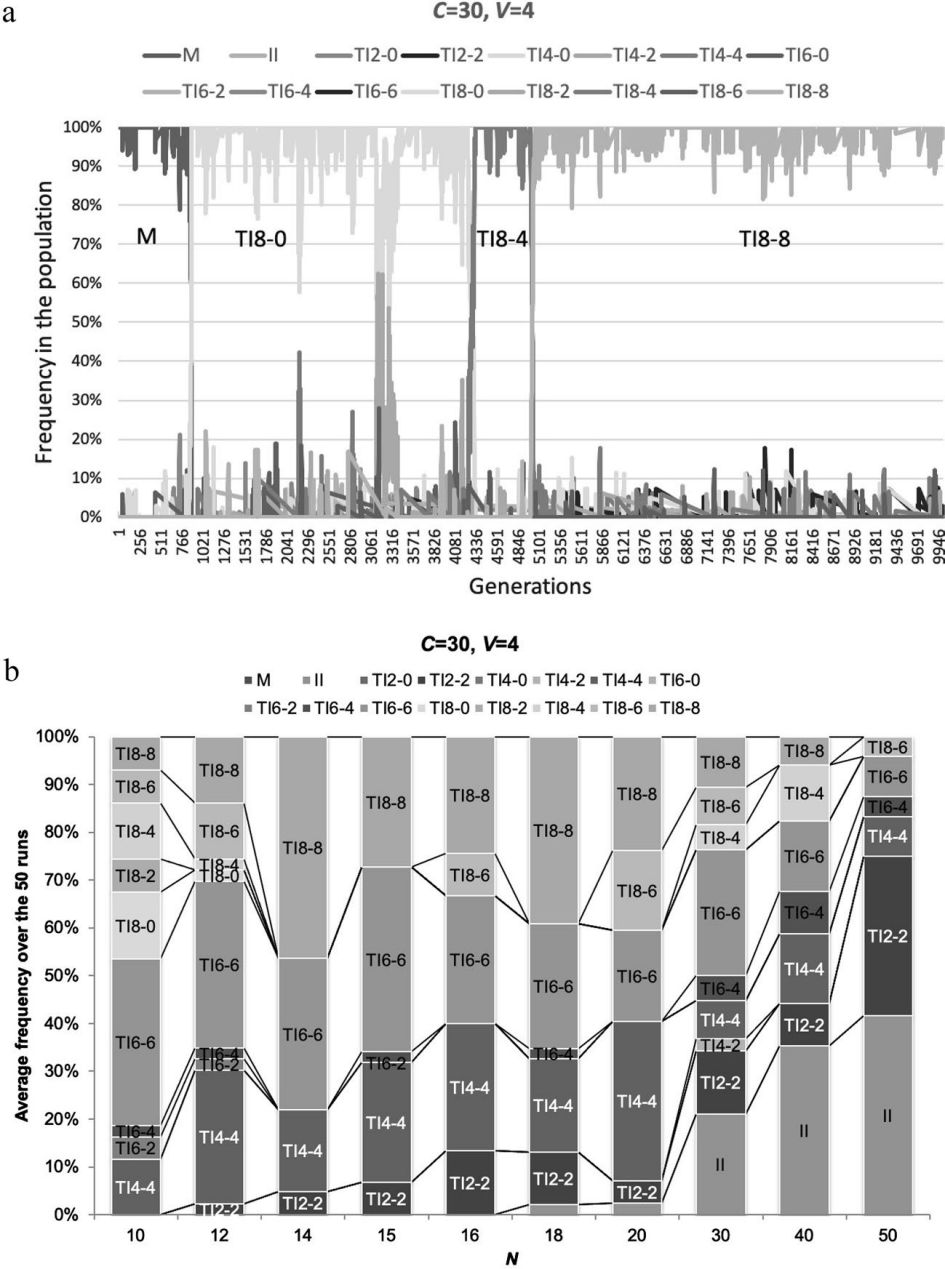

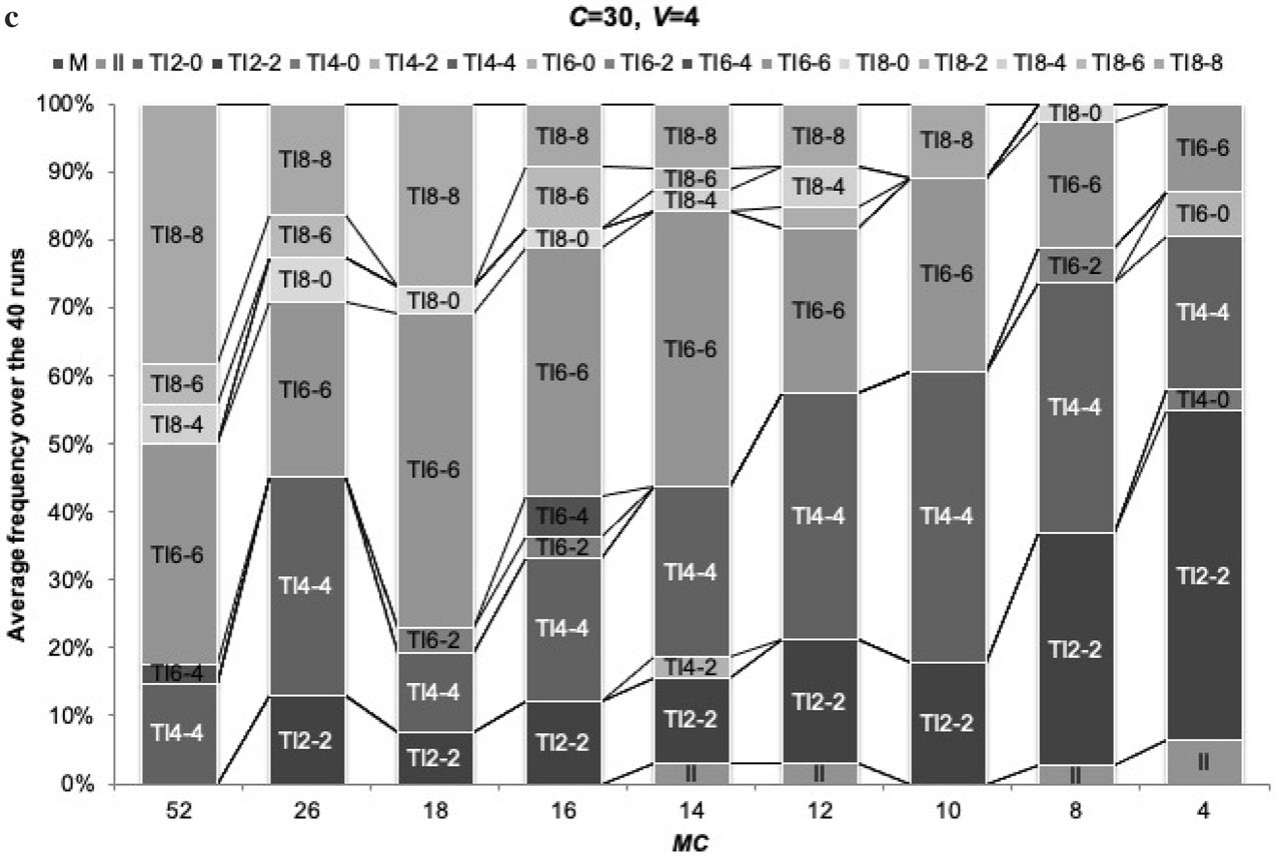
Evolutionary simulation with random mutation. We examined evolutionary dynamics of all strategies with mutation that occurs in two loci with a probability of *μ* (= 0.001) independently: one is for the number of reference members and the other is for the number of shared reference members. Initial strategy for all players is always a mixer strategy. Here we use the number of generations (*G*) = 10 000, *μ* = 0.001, *MC* = 14, *Np* = 2, *V* = 4 and *C* = 30. In a, b, *N* varies under the constant *MC*. **a** The vertical axis represents the final frequencies of strategies over generations (*G*) in a single run and the horizontal axis represents *G*. Here we use *N* = 20. **b** The vertical axis represents the final frequencies of dominant strategies as averages over 50 iterations and the horizontal axis represents *N*. The vertical axis indicates how often each dominant strategy appears in the 50 iterations. **c** Here we use the number of generations (*G*) = 10 000, *μ* = 0.001, *N* = 14, *Np* = 2, *V* = 4 and *C* = 30. *MC* (= 52, 26, 18, 16, 14, 12, 10 and 8) varies. In c, *MC* varies under the constant *N.* The vertical axis represents the final frequencies of dominant strategies as averages over 40 iterations and the horizontal axis represents *MC*. The vertical axis indicates how often each dominant strategy appears in the 40 iterations

We explored the evolutionary dynamics of strategies in various group sizes, assuming that an initial strategy for all players is a mixer strategy. We ran the evolutionary simulations with mutation with all 16 strategies over 10,000 generations, iterated 50 times, and calculated the average of population frequencies at each generation for each strategy. Each run out of 50 ends up with the dominant strategy without coexistence of strategies, as all strategies converges to a single strategy over the generations in a single run except a small number of mutants (Fig. 2a). A small number of mutants remains because mutation occurs randomly after one strategy dominates as designed.

Figure 2b shows the distribution of dominant strategies across different group sizes in the 50 iterations under the constant *MC.* Our analysis confirms that a group of reference transitive inference strategies is more dominant than the immediate inference strategy under limited cognitive abilities across any group sizes (Fig. 2b). More importantly, TI_*Z-Z*_ (*Z* = 2, 4, 6 and 8) strategies turn out to be more successful than other TI strategies, TI_*Z-Y*_ (*Y* < *Z*). We also note that immediate inference strategy becomes more successful when *N* ≥ 30. We will discuss the reason later in this section.

In addition, similarly to Fig. 2b, we looked into how different memory capacities (*MC*) impact the evolutionary dynamics of reference transitive strategies using the similar analysis applied in Fig. 2b where memory capacity (*MC*) is constant at 14 and the group size (*N* = 10, 12, 14, 15, 16, 18, 20, 30, 40 and 50) varies. In this *MC* sensitivity analysis, memory capacity (*MC* = 52, 26, 18, 16, 14, 12, 10, 8 and 4) varies and the group size (*N*) is constant at 14. It is, as expected, confirmed that a group of reference transitive inference strategies is more dominant than the immediate inference strategy under limited memory capacities and TI_*Z*–*Z*_ (*Z* = 2, 4, 6 and 8) strategies dominate TI_*Z-Y*_ (*Y* < *Z*) (Fig. 2c). Hereafter, our analysis is based on the framework of different group sizes (*Ns*) under the constant memory capacity (*MC* = 14).

**Fig. 3:**
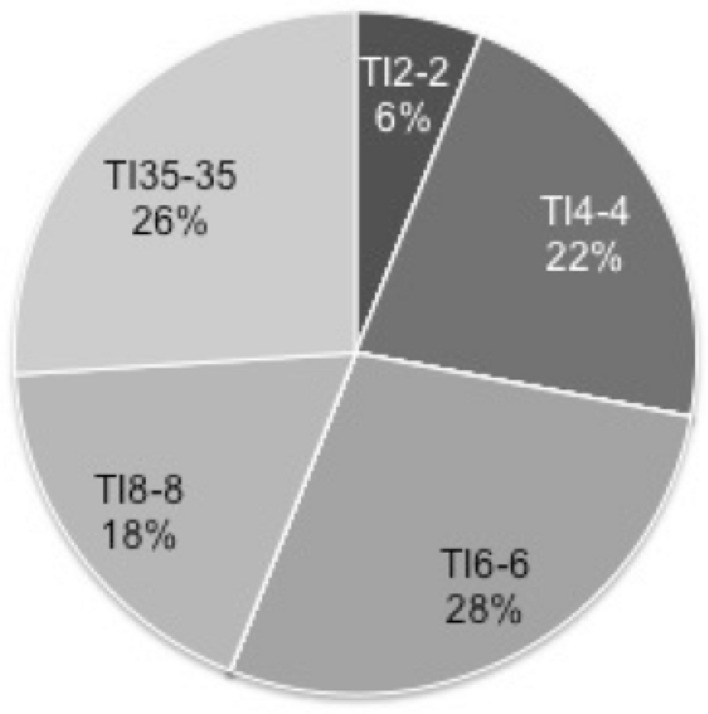
Evolutionary simulation without random mutation. We analyzed evolutionary dynamics of M, II, TI_2–2_, TI_4–4_, TI_6–6_, TI_8–8_ and TI_35–35_ with equal initial proportions under *N* = 35. No mutation is assumed. We ran the process 50 times and calculated the average frequency of each strategy. Each run ended with 100% of the most dominant strategy and there was no coexistence of strategies. Final strategy frequencies represent how often the respective strategies become the most dominant strategy. We calculated the averages of the final frequencies only when the survival strategy converged into a single strategy. Here *G* = 500, *MC* = 14, *Np* = 2, *V* = 4 and *C* = 30

Why, for example, is TI_4–4_ more successful than TI_8–2_ even though TI_8–2_ can recognize more group members? What if we introduce the standard transitive inference strategy, TI_*N-N*_ as TI_*N-N*_ can recognize all members. We looked into the evolutionary dynamics of strategies of M, II, TI_2–2_, TI_4–4_, TI_6–6_, TI_8–8_ and TI_35–35_ under *N* = 35 and *MC* = 14. We assume that all strategies start with equal initial frequencies and no mutation occurs over 500 generations and repeated it 50 times. Similarly, each run ends up with dominant strategy without coexistence of strategies. Average final frequencies in Fig. 3 mean how often each dominant strategy appears in the 50 iterations.

Results in Fig. 3 show that final frequencies of TI_4–4_, TI_6–6_, TI_8–8_ and TI_35–35_ are similar, and TI_2–2_ ends up with the smaller final frequency. Immediate inference strategy does not survive in Fig. 3. It is noteworthy that TI_35–35_, the standard transitive inference that can use information of all members in the group, does not make a meaningful difference from TI_*Z-Z*_ (*Z* = 4, 6 and 8). It is true that TI_*Z-Z*_ (*Z* = 4, 6, 8 and *N* is more successful than TI_2–2_ consistently both in Figs. 2b and 3. Results in Figs. 2b and 3 jointly suggest that the size of *Z* is not a key factor for survival as long as *Z* is greater than 2.

**Fig. 4:**
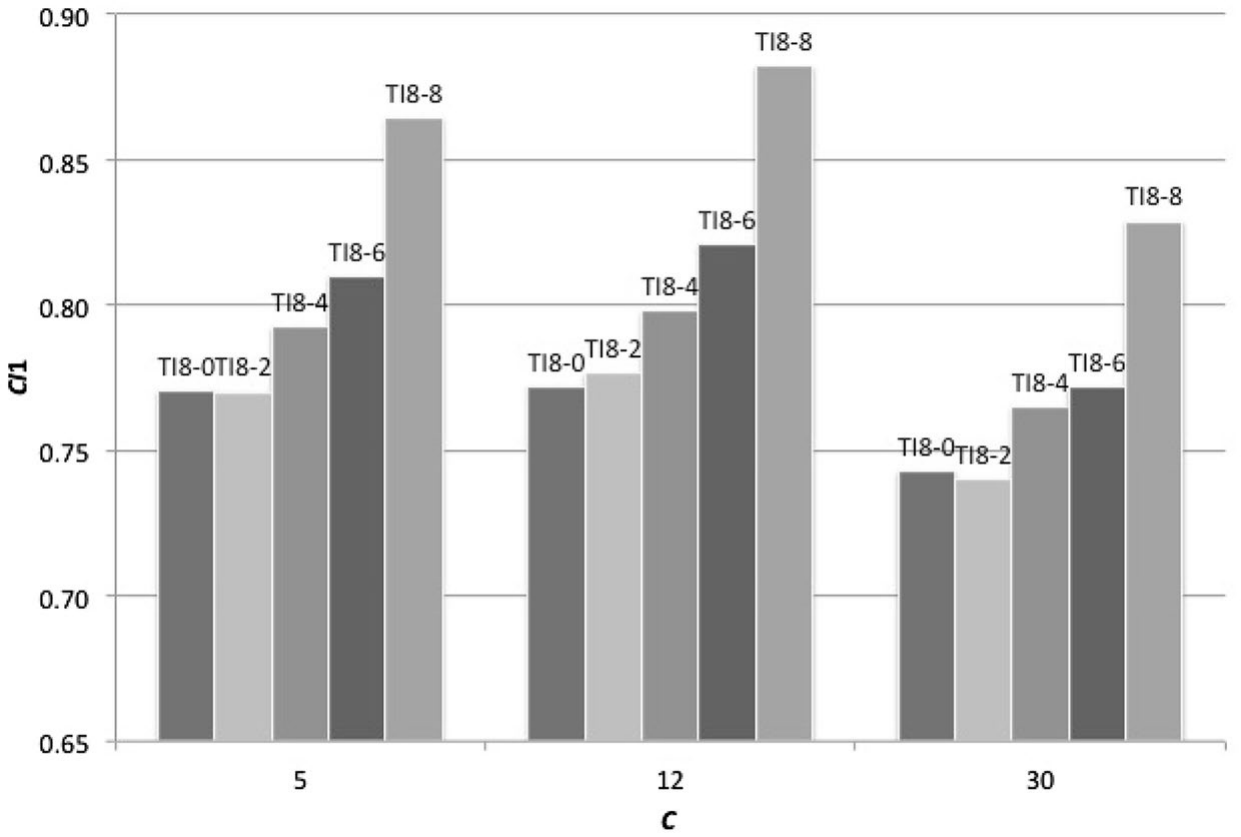
*CI* level based on strategies with a constant number of reference members and different numbers of shared reference members. We ran 240 games in one generation with *N* = 16 (*Np* = 2) and *MC* = 14. The horizontal axis indicates *C.* The vertical axis represents average *CI*1 indices over 100 iterations. Here *V* = 4, *C* = 5, 12 and 30

**Fig. 5:**
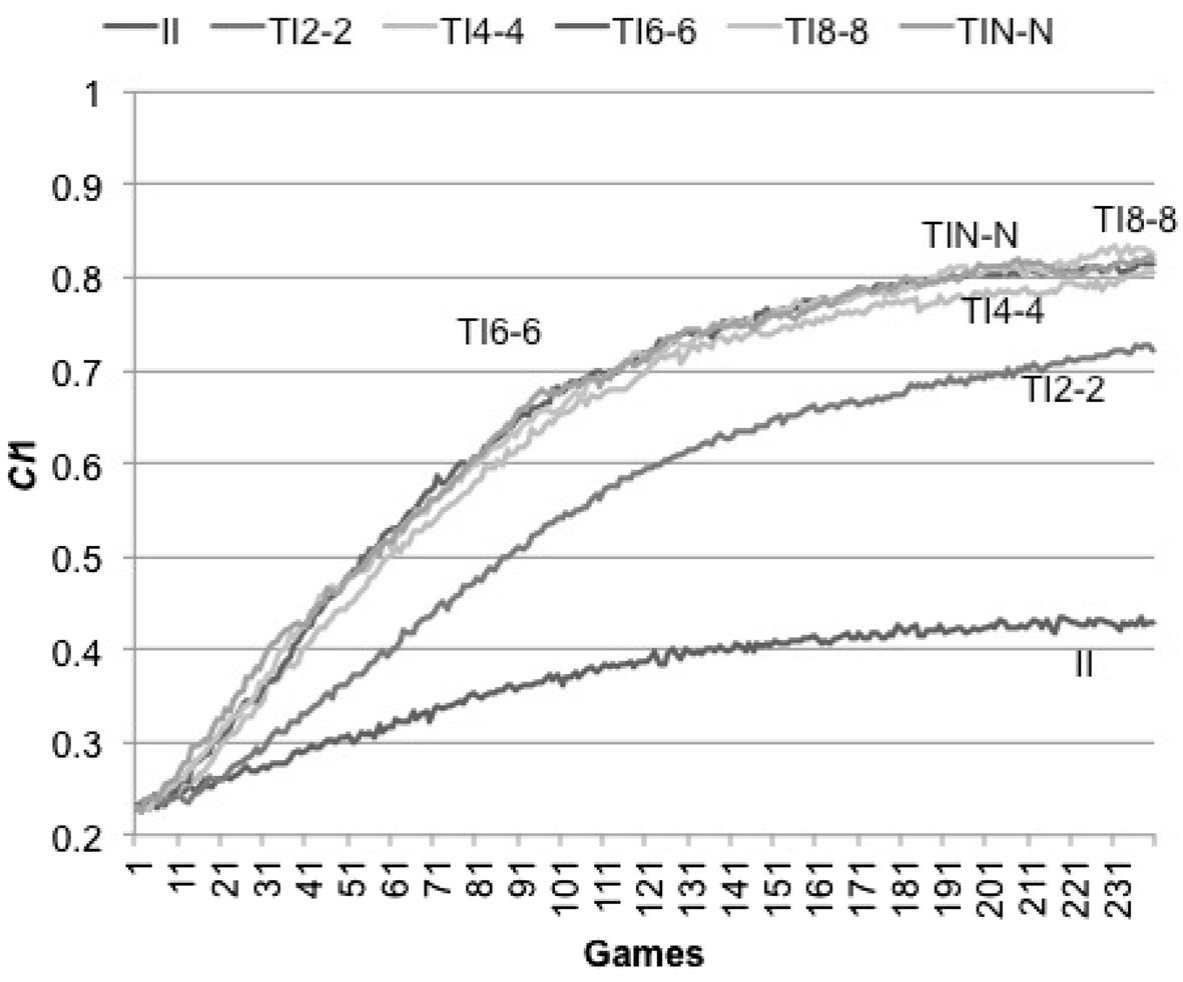
*CI*1 developments by strategies. We ran 240 games in one generation with *N* = 16 (*Np* = 2) and *MC* = 14. The horizontal axis indicates the number of games. The vertical axis represents averages of *CI*1 indices over 100 iterations. Line legend shows a strategy name. *C* = 30. Here *V* = 4

What is a key factor if the size of *Z* is not a key factor? Our hypothesis is that sharing more members (larger *Y*) is more critical than recognizing more members (larger *Z*) in forming the social hierarchy. By using the modified consistency index (*CI*_1_) explained in the model section, we examined how consistently social hierarchies are built in TI_*Z-Z*_ and TI_*Z-Y*_ (*Y* < *Z*) under *Z* = 8. Figure 4 demonstrates that TI_*Z-Z*_ strategies can form the social hierarchy better than TI_*Z-Y*_ (*Y* < *Z*) under any *C*. This result confirms our hypothesis that sharing reference members more with other members promotes the prompt establishment of the social hierarchy by using information from others’ experiences. The key factor to succeed is not the number of reference members (*Z*), but the number of shared reference members (*Y*) that is the ability to share the reference members (Fig. 4). In addition, Fig. 5 shows that TI_*Z-Z*_ (*Z* = 2, 4, 6, 8 and *N*) strategies can form the social hierarchy faster than immediate inference strategy. This promotes the evolution of transitive inference more in larger *C* where forming the linear social hierarchy is more important for survival.

Why does most TI_*Z-Z*_ strategies succeed similarly despite different *Z*? We examined how quickly the social hierarchies are built in II and TI_*Z-Z*_ (*Z* = 2, 4, 6, 8 and *N*) and found that *CI*_1_ in all TI_*Z-Z*_ (*Z* > 2) also develops indifferently (Fig. 5). We consider that similar *CI*_1_ behaviors are the reason for similar success in TI_*Z-Z*_ (*Z* > 2).

**Fig. 6:**
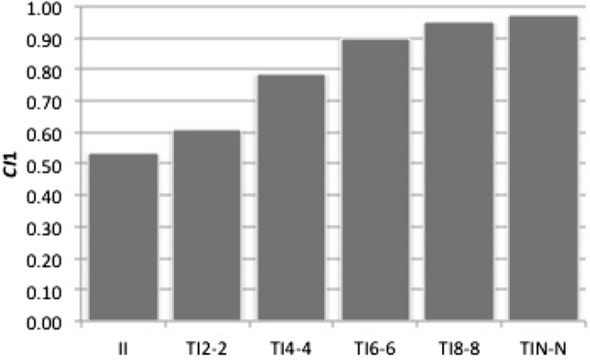
*CI*1 developments in TI_*Z-Z*_ with unlimited memory capacity. We ran 210 games in one generation with *N* = 15 (*Np* = 2) under unlimited memory capacity. The horizontal axis indicates a strategy name. The vertical axis represents averages of *CI*1 indices over 50 iterations. *Z* = 2, 4, 6 and 8. *C* = 30. Here *V* = 4

However, this finding seems a little counter-intuitive because higher *Z* should suggest higher cognitive abilities. We look into how *CI*_1_ in all TI_*Z-Z*_ (*Z* > 2) develops under unlimited memory capacity and confirm that *CI*_1_ with higher *Z* increases faster (Fig. 6). This means that limited memory capacity prevents TI_*Z-Z*_ with higher *Z* from being more successful. We find that all TI_*Z-Z*_ (*Z* > 2) including TI_*N-N*_, standard transitive inference, behaves similarly because of limited memory capacity. *Z* does not make a difference under restricted memory capacity relative to the group size.

Are TI_*Z-Z*_ strategies evolutionarily stable (ESS)? The evolutionary simulations beginning with all players applying TI_8–8_, TI_4–4_, or immediate inference end up with all players maintaining their respective strategies even at the end in a large group (*N* = 40) (Table 2). TI_8–8_, TI_4–4_ and immediate inference are all evolutionarily stable and could evolve if they are applied by the majority of a group. On the other hand, the evolutionary simulations beginning with all players applying TI_8–0_ or TI_4–0_ end with various combinations of final frequencies of different strategies (Table 2D and E). It is confirmed that TI_8-0_ and TI_4–0_ are not ESSs. In sum, TI_*Z-Z*_ (*Z* < group size (*N*)) is an ESS while TI_*Z-*0_ is not an ESS because TI_*Z-Z*_ shares reference members with others while TI_*Z-*0_ does not. As discussed earlier, the ability to share reference members is critical because it facilitates the prompt establishment of the social hierarchy (Figs. 4 and 5).

**Table 2:**
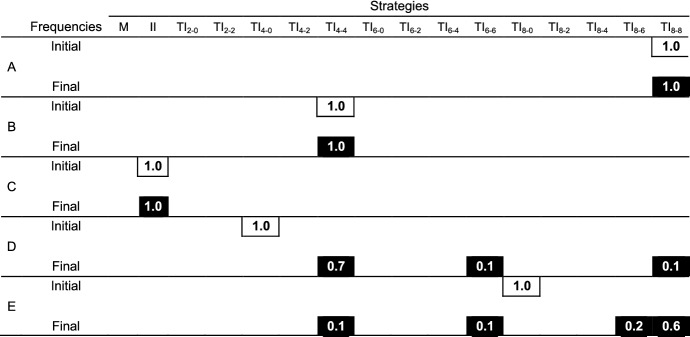
Evolutionary dynamics of all strategies with the random mutations

Evolutionary dynamics of all strategies with the random mutations that take place in two loci with a probability of *μ* (= 0.001) independently; one is for *x* and the other is for *y* in TI_*x–y*_. Each case, A, B, C, D, and E has a different initial strategy frequency. Initial strategy frequencies are as follows; A with TI_8–8_ = 100%, B with TI_4–4_ = 100%, C with II = 100%, D with TI_4–0_ = 100% and E with TI_8–0_ = 100%. Numbers in each cell represent the strategy frequencies at the start (upper row) and the end (lower raw) for each case, as averages over 50 times. Each run ends up with 100% of the most dominant strategies and no coexistence of strategies. Final strategy frequencies represent how often the respective strategies become the most dominant strategy. We calculate an average of final frequencies only when the survival strategy converges into a single strategy. The numbers in cells are rounded and sum of the numbers may not be 1 because of the rounding. We examine cases with two different *C*/*V* ratios (1.25 and 4). Here we use *N* = 40, *G* = 10 000, *μ* = 0.001, *MC* = 14, *C* = 30 and *V* = 4

**Fig. 7:**
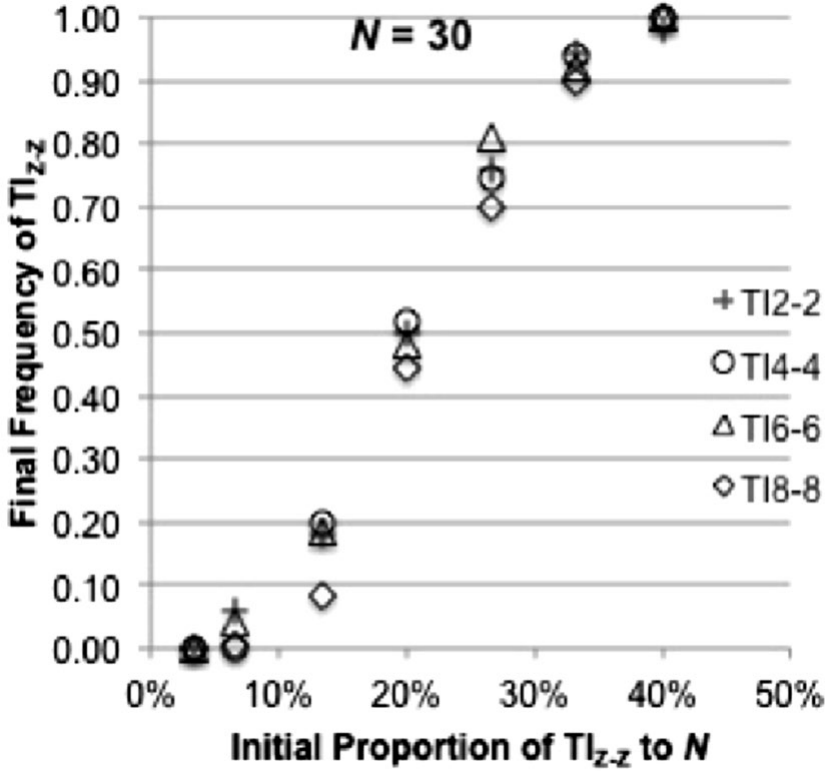
Influence of initial population on TI_*Z-Z*_. We analyzed evolutionary dynamics between II vs. TI with various initial proportions of TI_*Z-Z*_ under *N* = 30. *Z* = 2, 4, 6 and 8*.* The vertical axis represents the final frequencies of TI _*Z-Z*_ as averages over 50 iterations and the horizontal axis represents initial proportions of TI_*Z-Z*_ as % share of an entire population. Here *MC* = 14, *Np* = 2, *V* = 4 and *C* = 30

Finally, why does immediate strategy start to appear again and TI_*Z-Z*_ with higher *Z* begins to dominate less when the group size becomes very large (*N* ≥ 30) (Fig. 2b)? We consider that one of reasons is that the success of TI_*Z-Z*_ depends on initial proportions of strategies. TI_*Z-Z*_ with higher *Z* may require a higher initial proportion. We examined the evolutionary dynamics existing between immediate inference and TI_*Z-Z*_ under different group sizes to observe how final frequencies of TI_*Z-Z*_ develop over immediate inference with an increase in the group size (Fig. 7). No mutation was assumed. Figure 7 shows that TI_Z-Z_ strategies with higher (lower) initial proportions tend to result in higher (lower) final frequencies. This result suggests that TI_*Z-Z*_ has dependency on the initial proportions, meaning that TI_*Z-Z*_ requires a larger number of players following the same strategy to recognize the similar hierarchy.

This dependency hypothesis is confirmed by results in Fig. 2c because immediate inference never emerges under the constant group size. In addition, TI_*Z-Z*_ strategies survive over immediate inference in Fig. 3 where all strategies start with equal frequencies at the start, because TI_*Z-Z*_ is more likely to survive when the initial frequency of TI_*Z-Z*_ is greater (Fig. 7).

**Fig. 8:**
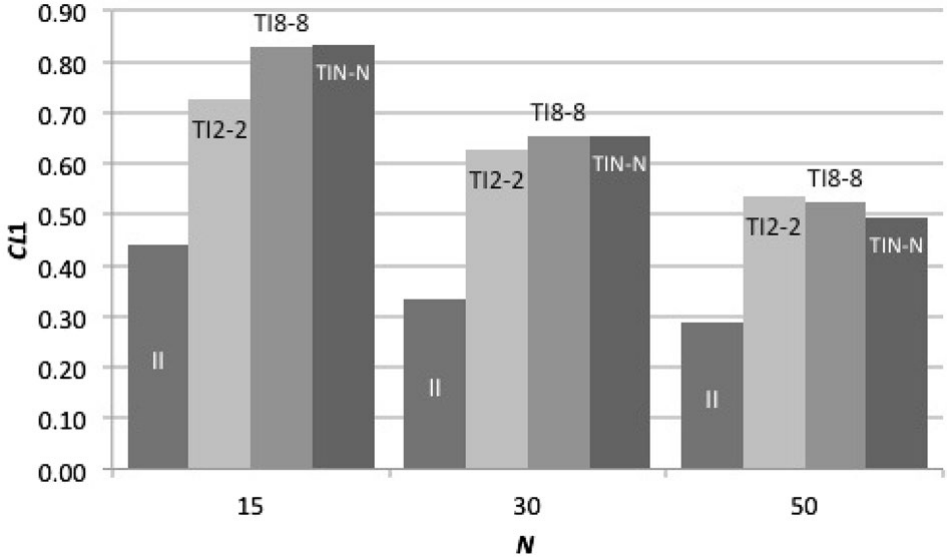
*CI*1 developments in TI_Z-Z_ under different group sizes. We ran 210 games in one generation with *N* = 15 (*Np* = 2) and with *MC* = 14. The horizontal axis indicates group size. The vertical axis represents averages of *CI*1 indices over 50 iterations for *N* = 15 and 30 and 30 iterations for* N* = 50. *Z* = 2, 4, 6 and 8. *C* = 30. Here *V* = 4

We consider that the other reason is that all strategies behave similar as *N* becomes very large (*N* ≥ 30). For example, the TI_8–8_ strategy with the largest cognitive abilities can observe, at the most, eight members in the group with more than 30 members. The TI_8–8_ strategy would not make a difference from the TI_2–2_ strategy that can observe only two members when the group size is very large. We looked into how *CI*_1_ between TI_2–2_ and TI_*Z-Z*_ (*Z* > 2) develop under different *N* and find that *CI*_1_ between TI_2–2_ and TI_*Z-Z*_ (*Z* > 2) becomes closer when *N* exceeds 30 (Fig. 8).

## Discussion and Conclusions

We looked into three possible different attributes of cognitive capability: 1) How many group members an individual can recognize (reference members), 2) How many reference group members an individual can share (shared reference members) and 3) How many contests in a group an individual can remember (memory capacity). We assumed that all three are limited relative to the group size. Doi and Nakamaru (2018) theoretically studied the impact of limited memory to the evolutionary dynamics of transitive inference and the social hierarchy formation.

We have shown that a group of reference transitive inference strategies is more dominant than the immediate inference strategy under the constraints of limited cognitive capacities in a large group. This suggests that the ability in transitive inference to observe interactions among others, even though it is limited, distinguishes transitive inference from immediate inference because transitive inference can form the social hierarchy much faster than immediate inference as the group size increases.

The other important finding of our study is that, within the reference transitive strategies, TI_*Z-Z*_ strategies dominate TI_*Z-Y*_ (*Y* < *Z*) strategies and TI_*Z-Z*_ strategies can persist even with the smallest *Z* across various group sizes (Fig. 2b and c). This is because sharing reference members more with other members (larger *Y*) promotes the prompt formation of the social hierarchy by using information from others’ experiences. The ability to share reference members (larger *Y*) is more important than the ability to broaden a set of reference members (larger *Z*) especially when memory capacity is limited (Fig. 2b and c).

While prior research has discussed the importance and evidence of transitive inference in the formation of the social hierarchy especially in a large group (Hotta et al. 2015; Mikolasch et al. 2013; Paz-Y-Miño et al. 2004; Tibbetts et al. 2022), the question kept nearly unanswered on how such seemingly cognitively demanding task as transitive inference can succeed as group size increases. Our study proposes a theoretical solution to the question: Use of heuristics such as reference transitive inference where reference members share individual memories.

The present study reveals that reference transitive inference is not necessarily cognitively demanding in terms of the number of reference members. However, reference transitive inference can evolve in a large group. This observation is potentially inconsistent with the idea that more highly developed social cognition needs to evolve as the group size increases because the larger group size increases social complexity substantially. Our study suggests that animals may apply a type of shortcut, or heuristics, in order to deal with increasing social complexity with an increase in the group size, instead of developing very high levels of social cognition.

On the other hand, our study also shows that reference transitive inference requires the ability to share individual memories through shared reference members. The ability to share individual memories is part of cognitive capability but this should be also part of collective memory related to communication and a network in a group (Coman et al. 2016; Weldon 2000). In this present study, we assumed the ability to share individual memories, but did not discuss how this ability is developed, how this ability relates collective memory and how a network structure in a group impacts the formation of collective memory. These remains open questions left for future discussions about transitive inference.
